# Concurrent pulmonary hypoplasia and congenital lobar emphysema in a young dog with tension pneumothorax: a rare congenital pulmonary anomaly

**DOI:** 10.1186/s13028-019-0472-2

**Published:** 2019-07-26

**Authors:** Hyun-Jung Han, Jung-Hyun Kim

**Affiliations:** 1 Department of Veterinary Emergency Medicine, Konkuk Veterinary Medical Teaching Hospital, Konkuk University, Seoul, 05029 Republic of Korea; 2 Department of Veterinary Internal Medicine, College of Veterinary Medicine, Konkuk University, Seoul, 05029 Republic of Korea

**Keywords:** Bronchial dysplasia, Dog, Emphysematous, Hypoplastic, Lung, Pneumothorax

## Abstract

**Background:**

Pulmonary hypoplasia (PH) and congenital lobar emphysema (CLE) are very rare congenital pulmonary anomalies in veterinary medicine. PH refers to the incomplete pulmonary development due to embryologic imbalance of bronchial development between the lung buds, while CLE is defined as alveolar hyperinflation due to bronchial collapse during expiration caused by bronchial cartilage dysplasia, external bronchial compression, and idiopathic etiology. CLE may develop into pulmonary blebs or bullae that may rupture and induce a spontaneous pneumothorax. There are no reports on concurrent PH and CLE in animals.

**Case presentation:**

A 7-month-old castrated male Italian Greyhound weighing 5.5 kg presented with vomiting and acute onset of severe dyspnea without any previous history of disease. After emergency treatment including oxygen supplementation and thoracocentesis, plain radiology and computed tomography scanning were performed and lobar emphysema with multiple bullae in the left cranial lung lobe associated with tension pneumothorax was identified. Since the pneumothorax was not resolved despite continuous suction of intrathoracic air for 3 days, a complete lobectomy of the left cranial lung lobe was performed. The excised lobe was not grossly divided into cranial and caudal parts, but a tissue mass less than 1 cm in size was present at the hilum and cranial to the excised lobe. Postoperatively, the dog recovered rapidly without air retention in the thoracic cavity. Histopathologically, the mass was identified as a hypoplastic lung tissue with collapsed alveoli, bronchial dysplasia, and pulmonary arterial hypertrophy. Additionally, the excised lung lobe presented CLE with marked ectasia of alveoli, various blebs and bullae, and general bronchial cartilage dysplasia. According to gross and histopathologic findings, the dog was diagnosed with concurrent PH and CLE in the left cranial lung lobe. During 16 months of follow-up, the dog was well and without any respiratory problems.

**Conclusions:**

This case report confirmed the clinical and histologic features of two different types of rare congenital pulmonary anomalies, PH and CLE, which occurred concurrently in a single lung lobe of a young dog. The condition was successfully managed with lobectomy.

## Background

Pulmonary hypoplasia (PH) and congenital lobar emphysema (CLE) are rarely reported congenital pulmonary anomalies in animals [1–5]. PH is a type of pulmonary agenesis that refers to the incomplete development of the pulmonary tissue [6]. It is classified into primary and secondary PH, that primary PH may result from lack of specific factors required for normal pulmonary development, and secondary PH is caused by other abnormalities such as abnormal thoracic cavity, abnormal fetal breathing movements, increased pressure of fetal lung fluid, and congenital heart disease with poor pulmonary blood flow [7]. In human medicine, it is suspected to be caused by the failure of the bronchial development to divide equally between the two lung buds, and predominately occurs in the left lung lobe than in the right lung lobe [6].

CLE is defined as alveolar air accumulation causing hyperinflation of the lung lobes due to abnormal dynamic airway collapse [8]. Dynamic airway collapse normally occurs at the end of the expiratory phase, but in CLE cases, it occurs at an improper time and manner that does not permit air elimination during expiration causing alveolar hyperinflation and increased expiratory intraalveolar pressure [8–11]. In humans, this pathophysiology of CLE was caused by three causes such as bronchial cartilage dysplasia leading to abnormal bronchial collapse during expiration, external bronchial compression from a deformed blood vessel, and idiopathic cases [4, 5]. This abnormal entrapment of air in the alveoli leads to progressive emphysema and development of pulmonary blebs or bullae that may rupture, inducing a spontaneous pneumothorax [9–11].

This study describes a canine case with two unusual congenital pulmonary anomalies (PH and CLE) in a single lung lobe with associated tension pneumothorax. To the best of the authors’ knowledge, this is the first report of two different types of congenital pulmonary anomalies occurring concurrently in a single lung lobe of a dog.

## Case presentation

A 7-month-old castrated male Italian Greyhound, weighing 5.5 kg was presented with vomiting and acute onset of severe dyspnea. According to the owner, the dog had been otherwise healthy without any relevant history. The dog had vomited several hours before attending the veterinary clinic, and subsequently, developed severe dyspnea after vomiting. On initial examination by the referring veterinarian, the dog was tachypneic (50 breaths/min). A large amount of air accumulation was observed in the left pleural space by radiographic evaluations done by the referring veterinarian. Left-sided thoracocentesis was performed with evacuation of 1.5 L of air from the thoracic cavity. After thoracocentesis, the dog was referred to the Veterinary Medical Teaching Hospital as an emergency case.

**Fig. 1:**
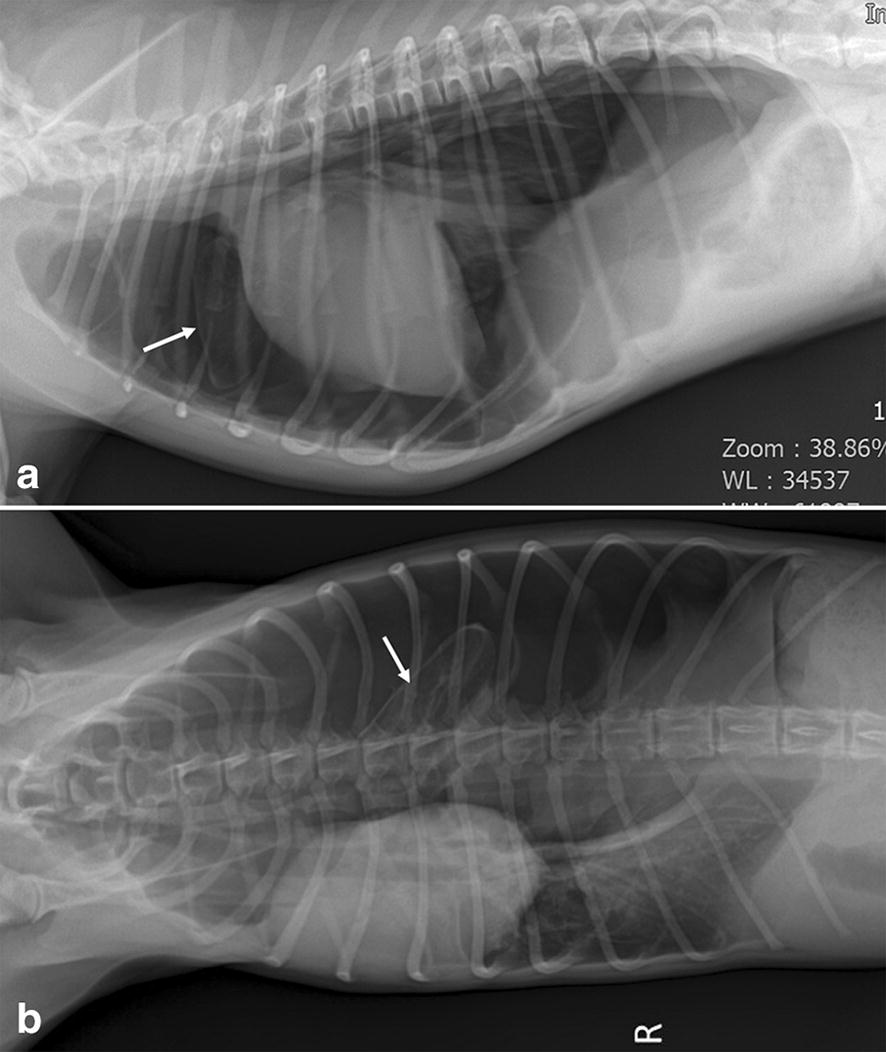
Right lateral (**a**) and dorsoventral (**b**) thoracic radiographs before emergent thoracocentesis. Large volume of air is retained in the left pleural cavity representing loss of sternal contact of the heart, gas in the left hemithorax, right mediastinal shift, and caudal displacement of the diaphragm with diaphragmatic tenting. The left cranial and caudal lung lobes are collapsed, while the left cranial lung lobe is hyperlucent with scant vascular markings (white arrows) compared to the other collapsed lung lobes

**Fig. 2:**
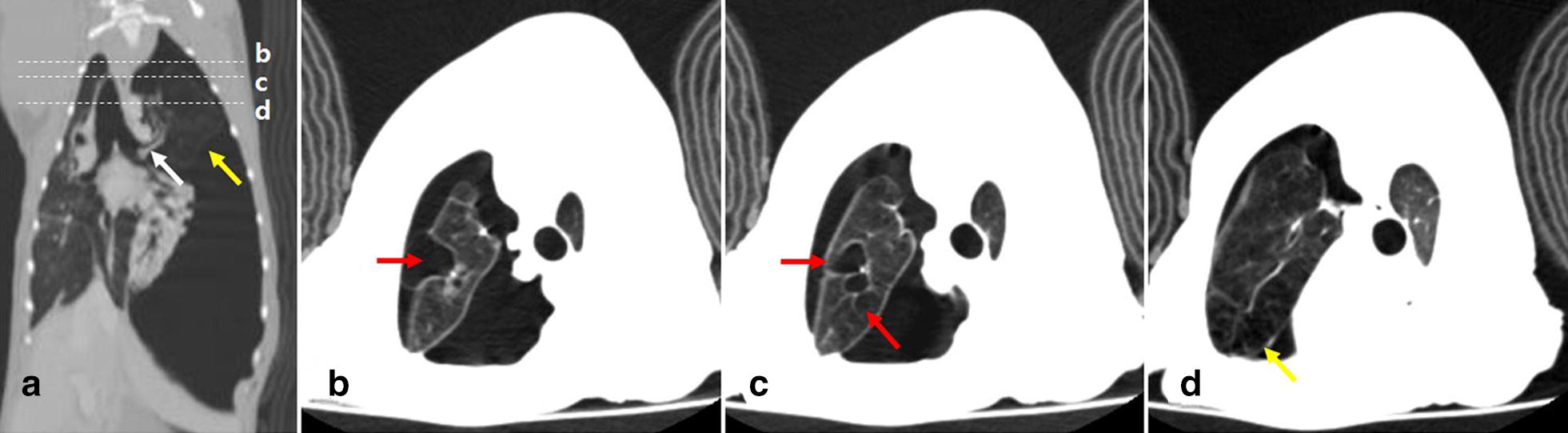
Post-contrast dorsal (**a**) and transverse (**b**–**d**) computed tomography scanning images of the thorax after emergent thoracocentesis. **a** Large volume of air is still identified in the left hemithorax. The left cranial lung lobe is hyperlucent and emphysematous with scant vascular markings (yellow arrows). Left cranial bronchus does not divide into cranial and caudal parts, and only the single lobar bronchus is identified in the left cranial lung lobe (white arrow). The white dotted lines and the lowercase letters b, c, and d represent the sections corresponding to the transverse images of **b**–**d** respectively. **b**–**d** Several round thin-walled pulmonary bullae of various sizes (red arrows) are observed in the hyperlucent and emphysematous left cranial lung lobe (yellow arrow)

On physical examination, the dog was alert and responsive with normal mucous membrane color and normal capillary refill time. The dog had a rectal temperature of 38.2 °C, pulse rate of 160 beats/min and a respiratory rate of 60 breaths/min showing labored breathing. The auscultation revealed indistinct heart and lung sounds on the left side of the thorax, but normal auscultation on the right side. Plain thoracic radiographs revealed the accumulation of a large volume of air in the left pleural cavity inducing displacement of diaphragm and mediastinum, and a hyperlucent left cranial lung lobe (Fig. 1). After emergency treatment including oxygen supplementation and thoracocentesis, computed tomography (CT) scanning was performed under general anesthesia and mechanical ventilation for further investigation. The CT images revealed the left-sided tension pneumothorax and emphysematous left cranial lung lobe with several bullae (Fig. 2). On CT scanning images, the left cranial lung lobe appeared as a single lobe with a single lobar bronchus that was not divided into cranial and caudal parts (Fig. 2a). Based on these findings, CLE and a ruptured pulmonary bulla were the most likely cause of the tension pneumothorax. After CT examination, a left-sided thoracostomy tube was placed, and continuous suction with a three-bottle system providing 10 to 15 cm negative pressure was applied for 3 days because of the rapid accumulation of air. Since the amount of air in the thoracic cavity did not decrease despite continuous suction, a surgical treatment was decided upon to remove the affected left cranial lung lobe.

**Fig. 3:**
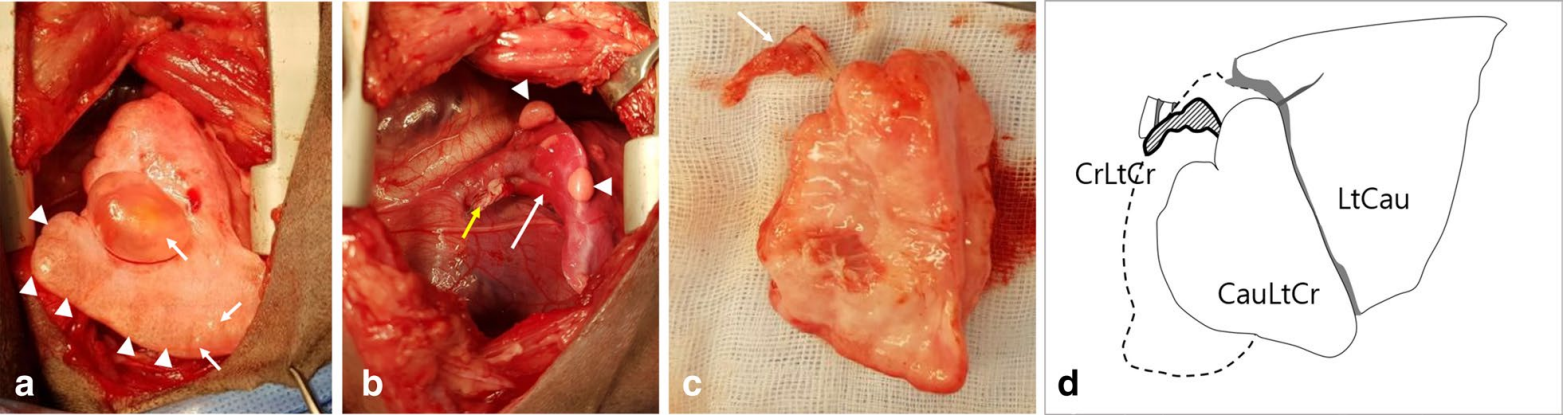
Intraoperative photograph of left fifth intercostal thoracotomy. **a** The left cranial lung lobe is overinflated with emphysema (white arrowheads) and small to large sized bullae (white arrows). **b** After removal of the left cranial lung lobe (yellow arrow), a totally collapsed left caudal lung lobe (white arrow) is identified, which re-inflated slowly (white arrowheads) after positive pressure ventilation. **c** The left cranial lung lobe is not divided, but a small tissue is identified at the cranial hilum of the excised lung (white arrow). **d** A line drawing illustration of the left lateral view of the lung shows that a normal size and shape of CrLtCr (dotted line) is not identified, and a hypoplastic lung is observed at the anatomical location of CrLtCr (bold solid line encircling gray hatching). (CrLtCr: Cranial part of left cranial lung lobe, CauLtCr: Caudal part of left cranial lung lobe, LtCau: Left caudal lung lobe)

The dog was premedicated with cefazolin (20 mg/kg intravenously [IV]), butorphanol (0.2 mg/kg IV), famotidine (0.5 mg/kg IV), and midazolam (3 mg/kg IV). After induction with propofol (4 mg/kg IV), the patient was intubated with an endotracheal tube and maintained with isoflurane (2%) in oxygen. Left fifth intercostal thoracotomy was performed in a routine fashion. The left cranial lung lobe appeared to be emphysematous with several small bullae and a large bulla, which was confirmed as the source of air leakage (Fig. 3a). A complete lobectomy of the left cranial lung lobe was performed using a thoracoabdominal stapler (DSTseries™ TA 30 mm Stapler, Covidien). The remaining left caudal lung lobe appeared to be collapsed, but its re-inflation was confirmed after positive end pressure ventilation (Fig. 3b). On inspection of the removed left cranial lung lobe, there was no division into cranial and caudal parts, but a tissue mass that was flat and less than 1 cm in size was attached to the hilum of the left lung lobe and located cranial to the left cranial lung lobe (Fig. 3c, d). Before closing the thoracotomy site, the thoracic cavity was filled with warm saline to detect any air leakage. A thoracostomy tube was placed and the intercostal thoracotomy was closed with 2-0 polydioxanone sutures around the ribs near the incision. Positive end pressure ventilation was maintained during the slow evacuation of the air from the thoracic cavity via the thoracostomy tube.

The dog recovered smoothly from anesthesia without any complications. Postoperatively, the dog exhibited a normal condition without dyspnea, and air retention was not identified in the thoracic cavity on postoperative radiographs. The radiographs revealed adequate expansion of the collapsed left caudal lung lobe. For postoperative analgesia, a continuous rate infusion of fentanyl (0.004 mg/kg/h) and lidocaine (1.2 mg/kg/h) was administered for 24 h postoperatively, followed by oral carprofen (2.2 mg/kg) and tramadol (4 mg/kg) twice daily for 7 days. The dog was discharged on the fifth postoperative day after removal of the thoracostomy tube. During 16 months of follow-up, the dog stayed well without any respiratory or radiographic abnormalities.

**Fig. 4:**
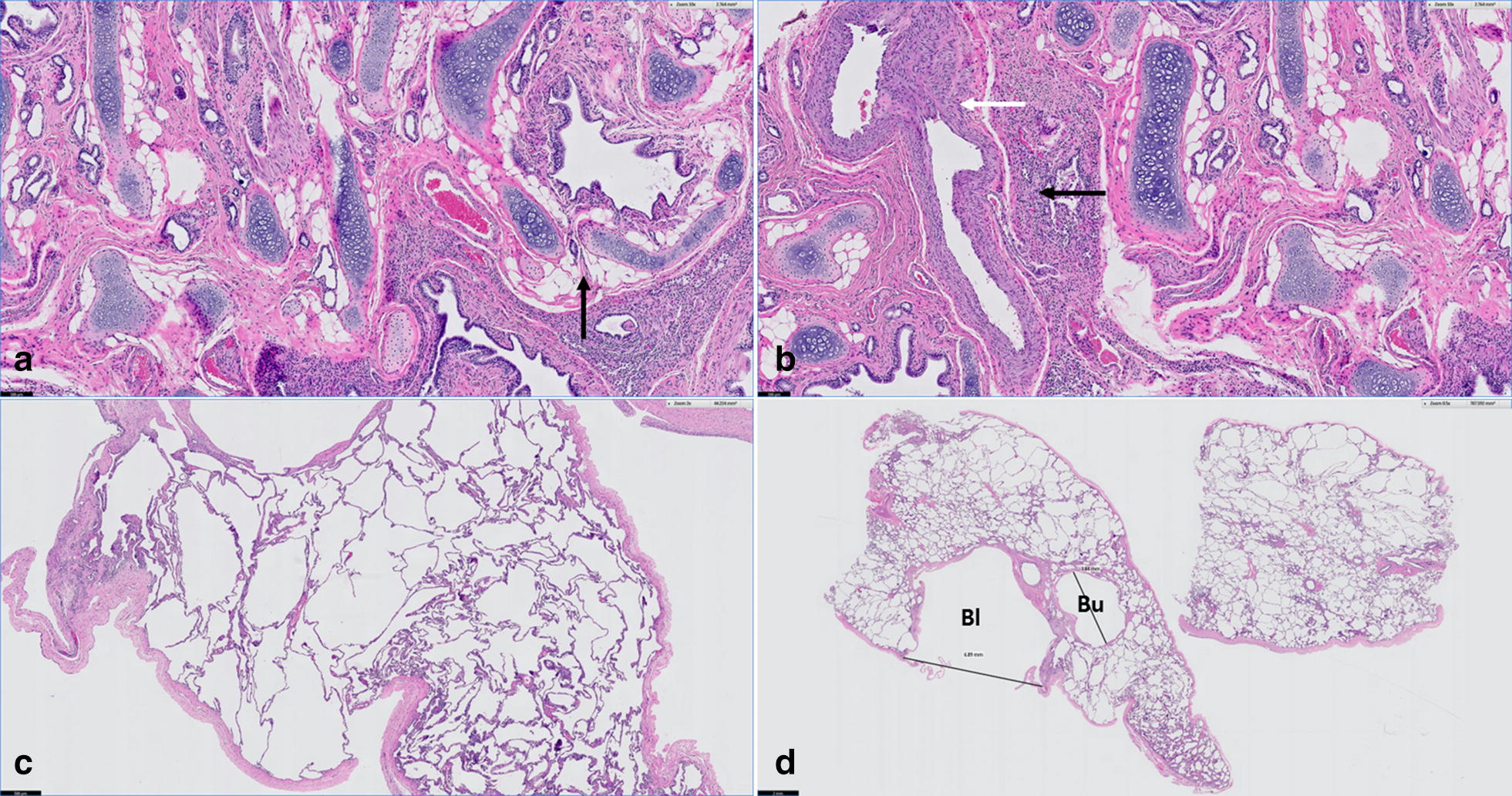
Photomicrograph of the excised left cranial lung lobe. **a**, **b** Small tissue mass attached to the excised lung: **a** disorganized cartilage plates with narrow underdeveloped airway lumens are observed. The arrow shows a tertiary bronchi with a dysplastic cartilage plates. Bar: 100 µm; **b** hypertrophic vessel (white arrow) and an area of bronchial cartilage dysplasia (black arrow). Bar: 100 µm. **c**, **d** The excised lung lobe: **c** Emphysematous alveoli and terminal bronchioles. Bar = 500 µm; **d** Bulla (Bu) and bleb (Bl) of approximately 4 and 7 mm in diameter were identified within the parenchyma and below the visceral pleura, respectively. Bar: 2 mm. **a**–**d** Hematoxylin and eosin stain

The excised left cranial lung lobe and tissue mass attached to it were histologically examined. The mass was identified as completely atelectatic lung tissue that was suspected to be the cranial part of the left cranial lung lobe (CrLtCr) based on its anatomical location. In the section, most alveoli had collapsed and bronchioles appeared to be somewhat dysplastic with normal columnar type epithelial cell lining. There were also areas of disorganized cartilage plate morphology and excessively smaller airways that may represent tertiary bronchi without adjacent cartilage plates (Fig. 4a). There was indications of hypertrophy of the medium-sized pulmonary arteries with vascular proliferation (Fig. 4b). The excised left cranial lung lobe, thought to be the caudal part of the left cranial lung lobe (CauLtCr), based on the anatomical location and shape, was characterized by the presence of emphysematous lung tissue, with marked ectasia of the alveolar lumens and terminal bronchioles and occasional formation of blebs and bulla (Fig. 4c). The classification of blebs and bullae was made by those location in the lung, that the bleb was found between the lung parenchyma and visceral pleura and the bulla was within the emphysematous parenchyma. Despite blebs is usually considered to be smaller than bullae, the diameters of the bleb and the bulla measured approximately 7 mm and 4 mm, respectively (Fig. 4d). This specimen also revealed that the cartilaginous plates lining the smaller or medium-sized bronchi appeared to be dysplastic and occasionally underdeveloped. According to the gross and histopathological findings, the excised pulmonary tissues were confirmed to have PH of CrLtCr and CLE of CauLtCr.

## Discussion and conclusions

PH can be diagnosed by gross findings, ratio of lung to body weight, and radial alveolar count, the number of alveoli bisected by the line drawn from the center of a respiratory bronchiole perpendicular to the nearest septal or pleural border [12, 13]. Particularly in human medicine, the objective guideline for using the weight of the lung and the histopathological measuring of the radial alveolar count was established for the diagnosis of PH in neonatal necropsy [13]. We could not equally apply the guideline for the diagnosis of PH in this case, because we could not weigh the entire lung in the living patient nor histologically measure the radial alveolar count due to lobar atelectasis leading to alveolar deflation. Instead, we diagnosed the tissue as PH based on gross and histopathological findings since the tissue size was small and represented characteristic histological features of PH including limited amounts of lung parenchyma, bronchioles and supporting vasculature, and hypertrophy of the pulmonary arterial walls [12]. CT scanning revealed the presence of the left cranial lung lobe appearing as a single lobe, without any cranio-caudal division; the hypoplastic lung was not visualized on CT. However, gross examination indicated the hypoplastic lung to be located cranial to the hilum of the excised left cranial lung lobe, and the anatomical location enabled its identification as the CrLtCr. Considering the young age of the dog and no obvious cause of secondary PH such as congenital abnormalities of lung, thoracic cavity, and heart, it was considered as primary PH, which could result from abnormal embryologic development due to idiopathic deficiencies in transcription factors or growth factors [6, 7]. In humans, developmental abnormality is assumed to be the cause of primary PH and in the failure of the bronchial development to divide equally between the two lung buds, eventually leading to disproportionate development of both lung lobes [6]. When PH occurs in isolated lung lobes rather than systemically, it is asymptomatic and does not require special treatment as reported in human and veterinary medicine [1, 6, 12]. In this case, it was found incidentally after resection of the left cranial lung lobe for the removal of the CLE and a ruptured bulla.

In the dog described here, the CauLtCr was confirmed affected by CLE with the presence of occasional blebs and bullae, and moreover, the affected lung lobe exhibited bronchial cartilage dysplasia. Bronchial cartilage dysplasia is one of the three pathological hallmarks of CLE in humans that has been presumed to be strongly associated with the development of CLE [4, 5]. Defective development of the bronchial cartilage may lead to bronchial collapse during expiration [8, 14]. This abnormal airway collapse traps air in the lower respiratory tract leading to lobar emphysema [8, 14]. Bronchial dysplasia is one of the most common histological finding of lung biopsy in veterinary and human cases of CLE [2–4, 15–17]. Bronchial cartilage dysplasia was also found in the hypoplastic CrLtCr.

The concurrent occurrence of PH and CLE in a single lobe of the lung may explain how two different congenital pulmonary anomalies triggered a potentially fatal tension pneumothorax in this dog. Hypoplastic lung lobes have been reported to cause compensatory hypertrophy of the contralateral lung lobes, indicated by increased airflow [1, 12]. In this case, PH of the CrLtCr could lead to compensatory hypertrophy of the CauLtCr, and the increased airflow would increase the accumulation of air at the level of alveoli, causing rapid progression of CLE and multiple blebs and bullae of various sizes to form, eventually leading to the accumulation of air between the visceral pleura and the lung parenchyma [18]. Concurrent occurrence of bleb and bulla suggests that alveolar hyperinflation may have occurred extensively throughout the entire lung lobe from the edge to the core of the pulmonary parenchyma. This broadly occurred alveolar hyperinflation is probably due to the bronchial dysplasia which is generally distributed in the lobe, and compensatory hypertrophy caused by PH may have aggravated the CLE and extensive bleb/bulla. Therefore, the concurrent occurrence of PH and CLE in a single lung lobe may have lead to extensive bleb/bulla formation which eventually induced a potential fatal spontaneous tension pneumothorax in the dog.

Tension pneumothorax should be treated immediately as it will otherwise be fatal due to hypoxemia and cardiovascular collapse [19]. Conservative treatment including emergent thoracocentesis and continuous suction of air from the pleural cavity through a thoracostomy tube can first be attempted for 2 to 3 days. If there is no response to the conservative treatment, lobectomy of the affected lung lobe is recommended for resolution [18, 19]. This patient was stabilized via immediate thoracocentesis followed by thoracostomy tube placement with continuous air evacuation. Despite continuous suction for 3 days, air continued to accumulate in the pleural cavity. Thus, complete lobectomy of the left cranial lung lobe was performed leading to complete resolution of the pneumothorax.

Both PH and CLE are rare congenital pulmonary anomalies in animals. This report presents the first case of these two anomalies occurring in a single lung lobe in a dog. In the dog described here, the cranial part of the left cranial lung lobe was hypoplastic, whereas, the caudal part was emphysematous and had bronchial cartilage dysplasia. PH of the cranial part was considered to induce compensatory hypertrophy of the caudal part possibly resulting in the aggravation of CLE and tension pneumothorax. The associated tension pneumothorax was successfully resolved with prompt emergency treatment and complete lung lobectomy.

## Data Availability

The datasets used and/or analysed during the current study are available from the corresponding author on reasonable request.

## Abbreviations

CauLtCr: caudal part of the left cranial lung lobe
CLE: congenital lobar emphysema
CrLtCr: cranial part of the left cranial lung lobe
CT: computed tomography
LtCau: left caudal lung lobe
PH: pulmonary hypoplasia

## References

[1] Lee CM, Kim JH, Kang MH, Eom KD, Park HM (2014). Unusual congenital pulmonary anomaly with presumed left lung hypoplasia in a young dog. J Small Anim Pract.

[2] Voorhout G, Goedegebuure SA, Nap RC (1986). Congenital lobar emphysema caused by aplasia of bronchial cartilage in a Pekingese puppy. Vet Pathol.

[3] Amis TC, Hager D, Dungworth DL, Hornof W (1986). Congenital bronchial cartilage hypoplasia with lobar hyperinflation (congenital lobar emphysema) in an adult Pekingese. J Am Anim Hosp Assoc.

[4] Herrtage ME, Clarke DD (1985). Congenital lobar emphysema in two dogs. J Small Anim Pract.

[5] Tennant BJ, Haywood S (1987). Congenital bullous emphysema in a dog. J Small Anim Pract.

[6] Roy PP, Datta S, Sarkar A, Das A, Das S (2012). Unilateral pulmonary agenesis presenting in adulthood. Respir Med Case Rep.

[7] Hsu JS, Lee YS, Lin CH, Li FY, Jeng MJ, Soong WJ (2012). Primary congenital pulmonary hypoplasia of a neonate. J Chin Med Assoc.

[8] Billet JP, Sharpe A (2002). Surgical treatment of congenital lobar emphysema in a puppy. J Small Anim Pract.

[9] Hoover JP, Henry GA, Panciera RJ (1992). Bronchial cartilage dysplasia with multifocal lobar bullous emphysema and lung torsions in a pup. J Am Vet Med Assoc.

[10] Gopalakrishnan G, Stevenson GW (2007). Congenital lobar emphysema and tension pneumothorax in a dog. J Vet Diagn Invest.

[11] Gelberg HB, McGavin MD, Zachary JF (2007). Alimentary system. Pathologic basis of veterinary disease.

[12] Kurkcuoglu IC, Eroglu A, Karaoglanoglu N, Polat P (2005). Pulmonary hypoplasia in a 52 year old woman. Ann Thorac Surg.

[13] Askenazi SS, Perlman M (1979). Pulmonary hypoplasia: lung weight and radial alveolar count as criteria of diagnosis. Arch Dis Child.

[14] Mawby DI, Krahwinkel DJ, Donnell RL, Morandi F (2006). Sebmental tracheal dysplasia in a mixed breed dog. Can Vet J.

[15] Karnak I, Senocak ME, Ciftci AO, Büyükpamukçu N (1999). Congenital lobar emphysema—diagnostic and therapeutic considerations. J Pediatr Surg.

[16] Ruth J, Rademacher N, Ogden D, Rodriguez D, Gaschen L (2011). Imaging diagnosis-congenital lobar emphysema in a dog. Vet Radiol Ultrasound.

[17] Orima H, Fujita M, Aoki S, Washizu M, Yamagami T, Umeda M (1992). A case of lobar emphysema in a dog. J Vet Med Sci.

[18] Lipscomb VJ, Hardie RJ, Dubielzig RR (2003). Spontaneous pneumothorax caused by pulmonary blebs and bullae in 12 dogs. J Am Anim Hosp Assoc.

[19] Puerto DA, Brockman DJ, Lindquist C, Drobatz K (2002). Surgical and nonsurgical management of and selected risk factors for spontaneous pneumothorax in dogs: 64 cases (1986–1999). J Am Vet Med Assoc.

